# Spontaneous posterior rectus sheath hernia: a case report

**DOI:** 10.1186/s13256-018-1645-8

**Published:** 2018-04-15

**Authors:** Chu Woon Ng, Anna Sandstrom, Grace Lim

**Affiliations:** Department of Surgery, Bundaberg Base Hospital, Bundaberg, QLD Australia

**Keywords:** Hernia, Rectus sheath, Abdominal wall, Case report

## Abstract

**Background:**

Hernias of the posterior rectus sheath are very rare abdominal wall hernias with only a handful of cases reported in the literature to date. As an uncommon disease, it is important to recognize and report this case in order to enhance scientific knowledge of this disease.

**Case presentation:**

This case report presents a spontaneous posterior rectus sheath herniation in a 79-year-old white man with previous abdominal surgery for appendicitis. His herniation was discovered incidentally during an examination for his chief complaints of lower abdominal pain and diarrhea which were later diagnosed as *Salmonella*-related gastroenteritis. A computed tomography scan of his abdomen and pelvis showed abdominal wall hernia with loops of small bowel extending into his rectus abdominis muscle. In this case, it was decided to leave the situation alone for now due to no evidence of bowel obstruction and the low risk of this hernia getting strangulated, which otherwise would have warranted urgent surgery.

**Conclusions:**

This report adds to the limited stock of available literature on this unusual issue and strengthens the evidence base on the best approach to support informed clinical decision making. The significant clinical implication of such case reports is increased identification rate of rare clinical conditions which otherwise often go unnoticed.

## Background

Hernias are an abnormal protrusion of an organ or tissue through a defect in its surrounding walls. A hernia can be congenital or acquired and the common types of hernia include inguinal, femoral, ventral, and incisional. Less common hernias include interparietal, Richter, and Littre hernias of the abdominal wall and sciatic, obturator, and perineal hernias in the pelvis [1].

Posterior rectus sheath hernias are a type of interparietal hernias where the hernial sac lies between the various layers of the abdominal wall muscles and does not exit into the subcutaneous tissue [1, 2]. The rectus abdominis muscle extends between the ribcage and pubis, and is supplied by the lower intercostal and corresponding segmental abdominal nerves. This muscle is enclosed within the rectus sheath which is formed by the aponeuroses of the lateral abdominal muscles [3].

This case report presents a summary of the case of a patient who presented with posterior rectus sheath hernia and discusses presenting features, diagnosis, and management in light of the current evidence. An informed consent has been obtained from the patient to present this case.

## Case presentation

A 79-year-old white man arrived by ambulance to our emergency department with chief complaints of lower abdominal pain and profuse diarrhea. On examination, general signs of poor health were observed. His blood pressure was 150/60 mmHg and pulse rate 90 beats per minute (bpm). His temperature was 37.3 °C and he did not appear to be in distress. A physical examination did not reveal any bowel obstruction. His laboratory findings were within normal range except for an elevated white blood cell count (14.5 × 10^9^/L), slightly low red blood cell count (3.12 × 10^12^/L), and slightly raised level of creatinine (124 μmol/L). His medical history included recent completion of radiotherapy treatment for a Gleason score 9 prostate adenocarcinoma, previous cerebrovascular event, asbestosis, atrial fibrillation, and chronic lymphoid leukemia. His abdominal surgical history included only an open appendectomy 40 years prior. He is a retired marine engineer and lives with his wife. He reported that he did not drink alcohol and had quit tobacco smoking 30 years ago. His family history is noncontributory.

**Fig. 1:**
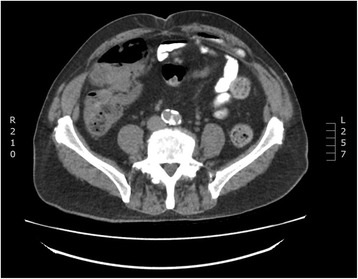
Computed tomography scan showing posterior rectus sheath hernia of a preperitoneal subtype

Computed tomography (CT) of his abdomen and pelvis was performed and demonstrated a peritoneal recess containing multiple small bowel loops over the anterior side of the intraperitoneum lying between peritoneal lining and the transverse abdominis fascia consistent with a pseudoherniation of a preperitoneal subtype (Fig. 1). There was no mass or dilatation of bowel loops and no mass to his upper abdominal organs or retroperitoneum.

In view of CT abdomen findings, a surgical opinion was sought. As there was no evidence of bowel compromise, he was managed non-operatively. Fecal polymerase chain reaction (PCR) tested positive for *Salmonella* and our patient improved on antibiotics with resolution of abdominal pain. He was discharged and followed up 2 weeks later at out-patient clinic and had no abdominal discomfort; therefore, no elective hernia repair was planned and further follow-up was not indicated.

## Discussion

Spontaneous posterior rectus sheath hernias were first documented in 1937. They are exceedingly rare with a case series in 2009 identifying only eight cases in the literature with two additional cases reported in 2014 and 2017 [4–6]. Among three different types of intraparietal hernias, interstitial is the most common [2, 3]. In our case, the hernia was preperitoneal as the hernia was located between the peritoneum and transversalis fascia [7].

To date, the pathophysiology of posterior rectus sheath hernia is not well understood [2, 6]. Examining the clinical anatomy of the abdominal wall, it is known that the rectus sheath is formed by interior oblique muscle, external oblique muscle, transversus abdominis muscle, and parietal peritoneum. This provides strong resistance against spontaneous herniation. However, below the arcuate line, the posterior rectus sheath comprises only transverse abdominis muscle which makes this structure weaker in comparison to the anterior sheath [6, 8]. Biomechanical analysis showed posterior sheath components of linea alba to be thinner than the anterior components which might be one possible explanation for internal structures invading a weaker structure [9]. In addition, it has been hypothesized that, similar to other type of hernias, any conditions which increase intra-abdominal pressure such as pregnancy, obesity, ascites, and progressive muscle weakness increase the likelihood of these hernias [3].

A majority of posterior rectus sheath hernia cases are post-surgical [5, 10] or post-traumatic. The remainder occur in persons without surgery [11] and are often reported as having a congenital origin [4]. In the literature, the age at diagnosis widely ranged from 25 to 83 years and posterior rectus sheath hernias were more common in women than in men [1, 6, 12]. Most hernias in the literature were reported as being approximately 2 cm in size, with the largest being 6 cm [4]. Clinical presentation can vary from abdominal pain, nausea, and vomiting to being relatively asymptomatic [2, 3, 5, 6].

Posterior rectus sheath hernia is usually diagnosed with imaging studies such as ultrasonography, magnetic resonance imaging (MRI), and CT. Out of these, CT is often considered the best diagnostic modality due to its higher sensitivity to highlight complications within the hernia sac such as obstruction, incarceration, and strangulation [1]. Laparoscopy has also been used for diagnostic and therapeutic purposes [12]. However, definitive diagnosis is often made during surgery [3]. Primary closure is the preferred choice of treatment with prosthetic repair in larger defects [6, 11]. Our case was managed conservatively due to no evidence of obstruction, ischemia, or strangulation, which would have warranted urgent surgery [3]. In the literature, there were cases in which patients were discharged after conservative management and only years later they had exploratory laparotomy for hernia repair [4].

## Conclusions

In our case, posterior rectus sheath hernia was an incidental diagnosis with no significant clinical course and was thus managed conservatively. Posterior rectus sheath hernias are uncommon and they may present with severe abdominal pain and incarceration, which can lead to an increased risk of bowel obstruction and strangulation if left untreated. Prompt diagnosis with or without surgery is needed for a successful outcome. This case report adds to the limited stock of available literature on this unusual issue and strengthens the evidence base on the best approach to support informed clinical decision making. The significant clinical implication of such case reports is increased identification rate of rare clinical conditions which otherwise often go unnoticed.

## References

[1] Aguirre DA, Santosa AC, Casola G, *et al.* Abdominal Wall Hernias: Imaging Features, Complications, and Diagnostic Pitfalls at Multi–Detector Row CT. Radiographics. 2005;25:1501–20. 10.1148/rg.256055018 16284131

[2] Felfel M, El Khoury M, Marboeuf Y, Strohl D, Menu Y. Incarcerated hernia through the posterior rectus sheath. AJR Am J Roentgenol. 2005;185:1185–6. 10.2214/AJR.04.0637 16247130

[3] Reznichenko A. Case of Rare Abdominal Wall Hernia. J Curr Surg. 2014;4(3):99–100.

[4] Lenobel S, Lenobel R, Yu J. Posterior Rectus Sheath Hernia Causing Intermittent Small Bowel Obstruction. J Radiol Case Rep. 2014;8(9):25–9. 10.3941/jrcr.v8i9.2081. PMC4242124 25426248

[5] Sogani J, Hentel KD, Belfi L. Emergency Imaging: Abdominal Pain 6 Months After Cesarean Delivery. Emerg Med. 2017;49(2):89-91.

[6] Losanoff JE, Basson MD, Gruber SA. Spontaneous hernia through the posterior rectus abdominis sheath: case report and review of the published literature 1937-2008. Hernia. 2009;13(5):555–8. 10.1007/s10029-009-0481-6 19234662

[7] Castillo-Sang M, Gociman B, Almaroof B, Fath J, Cason F. Non-traumatic lateral abdominal wall hernia. Hernia. 2009;13:317–21. 10.1007/s10029-008-0437-2 18949442

[8] Johnson TG, Von SJ, Hope WW. Clinical anatomy of the abdominal wall: hernia surgery. OA Anatomy. 2014;2(1):3.

[9] Korenkov M, Beckers A, Koebke J, Lefering R, Tiling T, Troidl H. Biomechanical and morphological types of the linea alba and its possible role in the pathogenesis of midline incisional hernia. Eur J Surg. 2001;167:909–14. 10.1080/110241501753361596 11841081

[10] Gangi S, Sparacino T, Furci M, Basile F. Hernia of the posterior lamina of the rectus abdominis muscle sheath: report of a case. Ann Ital Chir. 2002;73:335–7. 12404903

[11] Whitson BA, Ose KJ. Spontaneous posterior rectus sheath hernia: a new clinical entity? Hernia. 2007;11:445–7. 10.1007/s10029-007-0204-9 17297567

[12] Ahmed AR, Sharma A, Wellwood J. An unusual abdominal wall hernia. Ann R Coll Surg Engl. 2005;87(5):W1–2. 10.1308/147870805x50735 PMC1963981 16402456

